# Effects of vaccination on acute-phase protein response in broiler chicken

**DOI:** 10.1371/journal.pone.0229009

**Published:** 2020-02-11

**Authors:** Arash Janmohammadi, Nariman Sheikhi, Hadi Haghbin Nazarpak, Gholamreza Nikbakht Brujeni

**Affiliations:** 1 Department of Clinical Science, Faculty of Veterinary Medicine, Science and Research Branch, Islamic Azad University, Tehran, Iran; 2 Department of Clinical Science, Faculty of Veterinary Medicine, Garmsar Branch, Islamic Azad University, Tehran, Iran; 3 Department of Microbiology and immunology, Faculty of Veterinary Medicine, University of Tehran, Tehran, Iran; Macfarlane Burnet Institute for Medical Research and Public Health, AUSTRALIA

## Abstract

Broiler chickens experience an acute-phase response (APR) through vaccination, which reflects the innate immunity and stress related to immunization. It is also considered that APR can modulate adaptive immunity and response to infection. As biomarkers for APR, assessing the acute-phase proteins (APPs) function and their levels in response to immunization is of great value for vaccine design, development and administration. In this study, the heterophils/lymphocyte (H/L) ratio and the level of APPs was evaluated in broilers with three different Newcastle disease (ND) vaccination regimens. Inactivated ND vaccine (IND) was administered by the intramuscular route. Live attenuated strains, Lasota and Vitapest, was administered by ocular routes. H/L ratio, serum amyloid A (SAA) and alpha-1 acid glycoprotein (AGP) were measured before and after two rounds of vaccination on days 10 and 21. In a comparison between the three vaccines, H/L ratio in IND group significantly increased to 3 fold (1.48 ± 0.41) after the first vaccination while the Lasota and Vitapest showed a milder response. The concentration of SAA increased after 24h by 1.8-fold in IND group (0.116 ± 0.015 mg/L) and 2-fold in Lasota group (0.14 ± 0.002 mg/L). Significant changes were found in Vitapest group after 48h post vaccination (0.113 ± 0.016 mg/L). Elevation pattern of AGP, 24 hours after first vaccination in IND (3.5-fold) and Vitapest (2.5-fold) was different from Lasota in which the peak was reached after 48 hours (2.9-fold). Except for IND group, no significant changes in SAA and AGP concentrations were detected after the second vaccination. A significant positive correlation between SAA values at day 22 and HI titers at day 28 (r = 0.998, P≤0. 0.005) was found. According to these results, different types of ND vaccines can cause different patterns of acute phase responses. Assessment of stress and level of acute-phase proteins can be used for prediction of immune response outcomes in vaccine design and development.

## Introduction

Acute phase proteins (APPs) are major components of innate immune response to infection, stress and trauma. APPs are mainly produced in liver and their levels in blood largely reflect the inflammatory states. Serum concentrations of these proteins increase (positive APPs) or decrease (negative APPs) during acute-phase response (APR) [1]. In veterinary diagnostics APPs are used as biomarkers for inflammatory disease monitoring, responding to antibiotic or steroid therapy and animal production [2].

Adaptive immunity and immune memory are modulated by numerous factors and mediators in early innate immunity. Although the importance of this regulation is well documented, the role of APR has been unclear. APR refers to the part of innate reactions that can be measured by inflammatory biomarkers such as APPs. Understanding the APPs functions and their changing levels in respond to immunization is of great value for vaccine design, development and administration. In response to vaccination, APPs, as reliable biomarkers, can be used for predicting the immune memory and vaccine efficacy. Effects of vaccination on acute-phase response (APR) has been investigated in human [3], cattle [4], horses [5], piglets [6], fish [7] and chicken [8].

Researches on broiler chickens have shown the effects of feed deprivation, environmental temperature and stocking density on levels of APPs [9,10]. Kaab et al. (2018) also investigated the influence of vaccination against Newcastle disease (ND) and infectious bronchitis virus on level of alpha-1 acid glycoprotein (AGP) and serum amyloid A (SAA) in a group of specific pathogen free (SPF) layer chicks. SAA is a major, and AGP is a moderate positive APP that known to reflect the APR in chickens [8]. To the best of our knowledge there exists no data demonstrating APPs in the broiler chicken in response to vaccination, and possible variations between the administrations of different vaccines.

In this study, we followed the same protocols used by Kaab et al. (2018) to evaluate APR in commercial broilers chicken. We measured the AGP, SAA, and the heterophils/lymphocyte (H/L) ratio in responses to three ND vaccines including an inactivated oil emulsion vaccine used by intramuscular route and two live attenuated vaccines used by ocular route.

## Materials and methods

### Birds housing and vaccination

The study population consisted of 150 one-day-old Ross 308 broiler chicks, obtained from a local hatchery. All chicks were housed and fed with commercial basic feed according to Ross 308 farming standard protocol. On day 1, the chicks were randomly allocated to five experimental groups. Three groups of equal number of birds, 30 per treatment and 10 per replicate pen, were subjected to vaccination against the Newcastle disease. Two groups having the same number of birds (n = 30, 10 per replication) were kept as control groups.

Vaccination schedule was carried out as used locally. On days 10 and 21, the first group was intramuscularly administered inactivated oil emulsion of ND vaccine (IND) (CEVAC Broiler ND K, Hungary). Vaccine contained inactivated La Sota strain of ND virus, with oil adjuvant and merthiolate as preservative. The second and third groups were respectively administered Lasota (Pestikal GENERA, Croatia) and Vitapest (CEVAC VITAPEST L, Hungary) vaccines by ocular route (eye drop application). The remaining two groups were the negative controls which were administered PBS by intramuscular (Con. IM) and ocular (Con. OC) routes.

### Blood sampling

Blood samples (1–1.5 mL per bird) were collected at 8 sampling time points: day-old chicks in order to measure the maternal derived antibody; before 1^st^ vaccination (d 9) and 1 and 2 days after vaccination (d 11 and d 12); before 2^nd^ vaccination (d 20) and 1 and 2 days after 2^nd^ vaccination (d 22 and d 23); 7 days after 2nd vaccination (d 28) in order to measure the antibody titer against the vaccines. The chicks were then kept until the end of their growing period (d 45) and sent to slaughterhouse.

### Assessment of acute phase proteins and heterophil/lymphocyte ratios

The enzyme-linked immunosorbent assay (ELISA) kits for chicken (Gallus) AGP and SAA were obtained from Cloud-Clone Corp. (TX, USA). Procedures for measurements were conducted according to manufacturer’s instructions. Heterophil/lymphocyte ratios were determined as previously described by Kaab et al. (2018) [8].

### Assessment of antibody titers against the vaccine

Antibody titer against ND vaccines was measured using Haemagglutination-Inhibition (HI) test. Four haemagglutination units of antigen (HA) and two-fold diluted serum were used. HI antibody titers were expressed as reciprocal log2 values of the highest serum dilution causing complete inhibition of haemagglutination. One day old chicks were sampled for estimating the maternally derived antibodies and 7 days after second vaccination (28 days old) for antibody response to vaccines.

### Statistical analysis

The SAA, AGP, H/L ratios and antibody titer of the vaccinated and control groups, were expressed as means, and standard errors of the means (SEM). Significant differences between two groups were analyzed by the unpaired two-tailed Student’s t-test and for multiple mean comparisons, analysis of variance (ANOVA) and post-hoc tests. Repeated-measures ANOVA was used to test for significant differences in different sampling time points. Statistical significance is considered as P≤0.05. Fisher’s r-to-z conversion of correlation coefficients was used to obtain the *P* values in correlation analysis between HI titers and APR.

### Ethical issues

The ethical committee of Islamic Azad University approved the study (Ethical No. IR.IAU.SRB.REC.1397.4.9).

## Results

The heterophil/lymphocyte ratios were compared between three vaccine groups (Table 1). One day after the first vaccination, a highly significant (P < 0.01) increase in H/L ratios was observed in all vaccinated groups when compared to the pre-vaccination time (Table 1). In multiple comparisons, a highly significant difference (P <0.001) was noted for IND vs. Con. IM (Mean Diff. 1.17) as well as significant differences (P <0.05) for Lasota vs. Con. OC (Mean Diff. 0.60). There was no significant difference observed between Vitapest and Con. OC (Mean Diff. 0.31). Significant differences (P <0.05) were also observed for IND vs. Lasota (Mean Diff. 0.54) and a highly significant differences (P < 0.001) for IND vs. Vitapest (Mean Diff. 0.29). There was no significant difference between IND and Lasota ratios after treatments.

**Table 1:** Shows H/L ratio, SAA and AGP levels for vaccinated and control groups at pre-vaccination and day-1 after vaccination. All data are expressed as mean ± SD, n = 10 birds per sampling time.

	H/L (ratio)		SAA (mg/L)		AGP (mg/L)	
	Pre-vac	Post-vac	Pre-vac	Post-vac	Pre-vac	Post-vac
IND	0.63±0.13	1.48±0.41	0.051±0.014	0.102±0.019	368.2±152.1	1082.3±295.1
Con. IM	0.44±0.12	0.51±0.06	0.062±0.011	0.069±0.010	143.8±120.5	374.8±168.7
Lasota	0.43±0.12	1.23±0.47	0.065±0.015	0.114±0.015	389.3±161.2	528.2±271.6
Vitapest	0.65±0.17	0.94±0.48	0.083±0.01	0.100±0.014	487.9±206.3	531.5±460.1
Con. OC	0.71±0.13	0.63±0.08	0.072±0.012	0.066±0.019	175.5±97.1	354.1±176.8

The level of SAA was compared between vaccine groups (Table 1). SAA concentrations were significantly (P < 0.001) higher in all vaccinated groups when compared to pre-vaccination level (Table 1). At day-1 post first vaccination, SAA was also significantly higher (P <0.05) in all vaccinated groups compared to the controls (Mean Diff: IND vs. Con. IM, 0.03; Lasota vs. Con. OC, 0.05; Vitapest vs. Con. OC, 0.03). SAA levels were not statistically different between IND, Lasota and Vitapest vaccine groups.

There was no significant increase in AGP concentrations after vaccination except for IND group (P < 0.05) (Table 1). Also no significant difference was found between the ocular vaccines and the control groups. There was found a significant difference in the level of AGP in IND vaccinated group compared to Con. IM (Mean Diff. 835.6).

**Fig 1:**
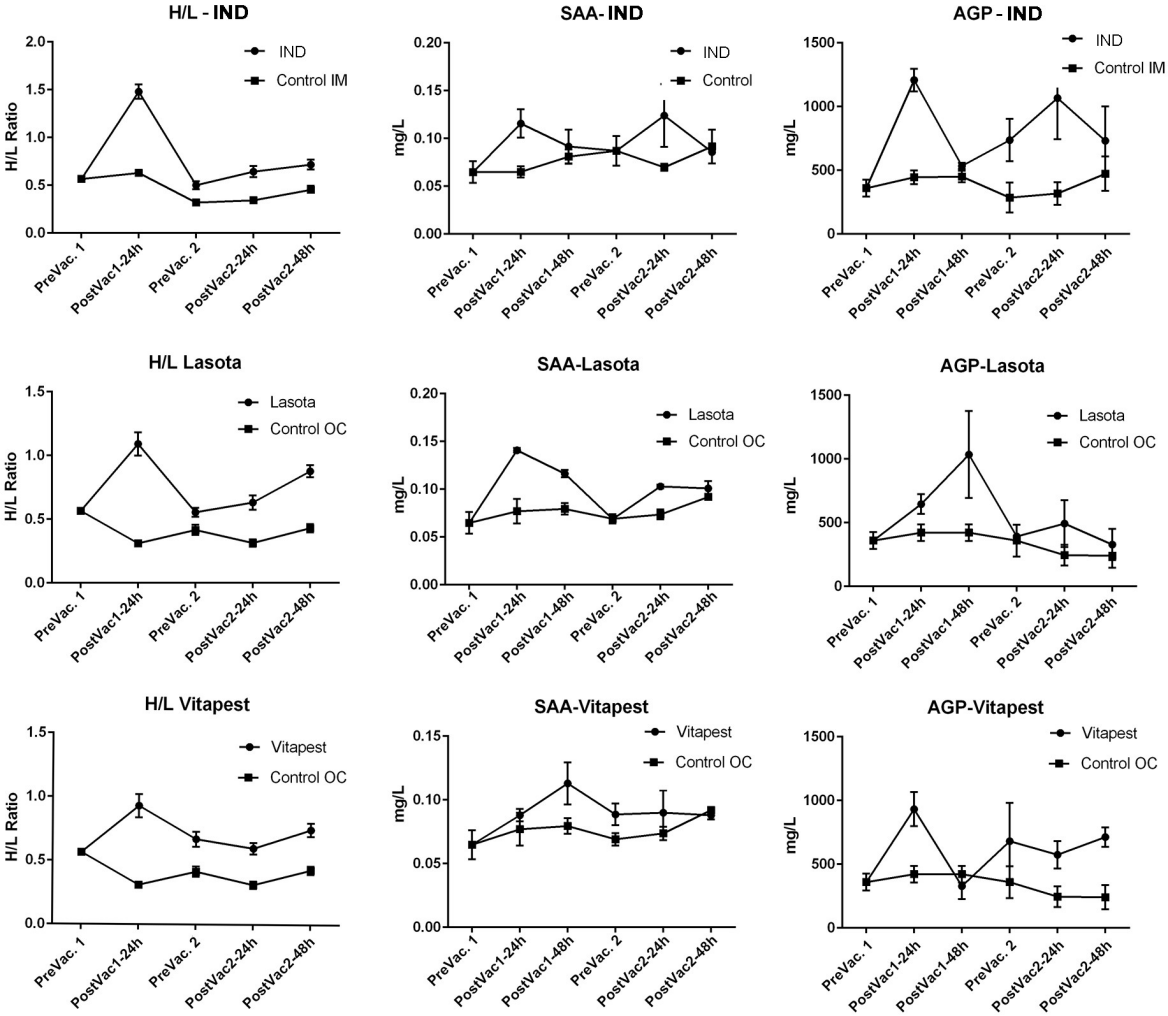
H/L ratio and level of acute phase proteins in responses to vaccination for each time point (n = 10 per time point). Data of sampling time points before first vaccination, day 9 (PreVac.1); after vaccination, days 11 (PostVac1-24h) and 12 (PostVac1-48h); before second vaccination, day 20 (PreVac.2) and after second vaccination, days 22 (PostVac2-24h) and 23 (PostVac1-48h) are presented.

H/L ratio and level of acute phase proteins in responses to vaccinations were compared with controls at each time points including: before first vaccination on day 10 (day 9), 1 and 2 days after first vaccination (days 11 and 12), before second vaccination on day 21 (day 20) and 1 and 2 days after second vaccination (days 22 and 23) (Fig 1).

After first vaccination, H/L ratios as well as SAA and AGP concentrations sharply increased in all vaccinated groups and then gradually decreased prior to the second vaccination time (Fig 1). Before second vaccination (Pre Vac.2), H/L ratios in all groups were statistically different from the controls. At the same time, SAA and AGP levels were not statistically different from the controls, except for SAA in Vitapest and AGP in IND groups. After second vaccination (Post Vac.2), IND and Lasota groups showed a significant increase in their SAA and AGP levels. After second vaccination by Vitapest no significant changes was observed for SAA but the level of AGP was significantly higher than control (Control OC) and stayed in different level until the end of the experiment (Fig 1).

**Fig 2:**
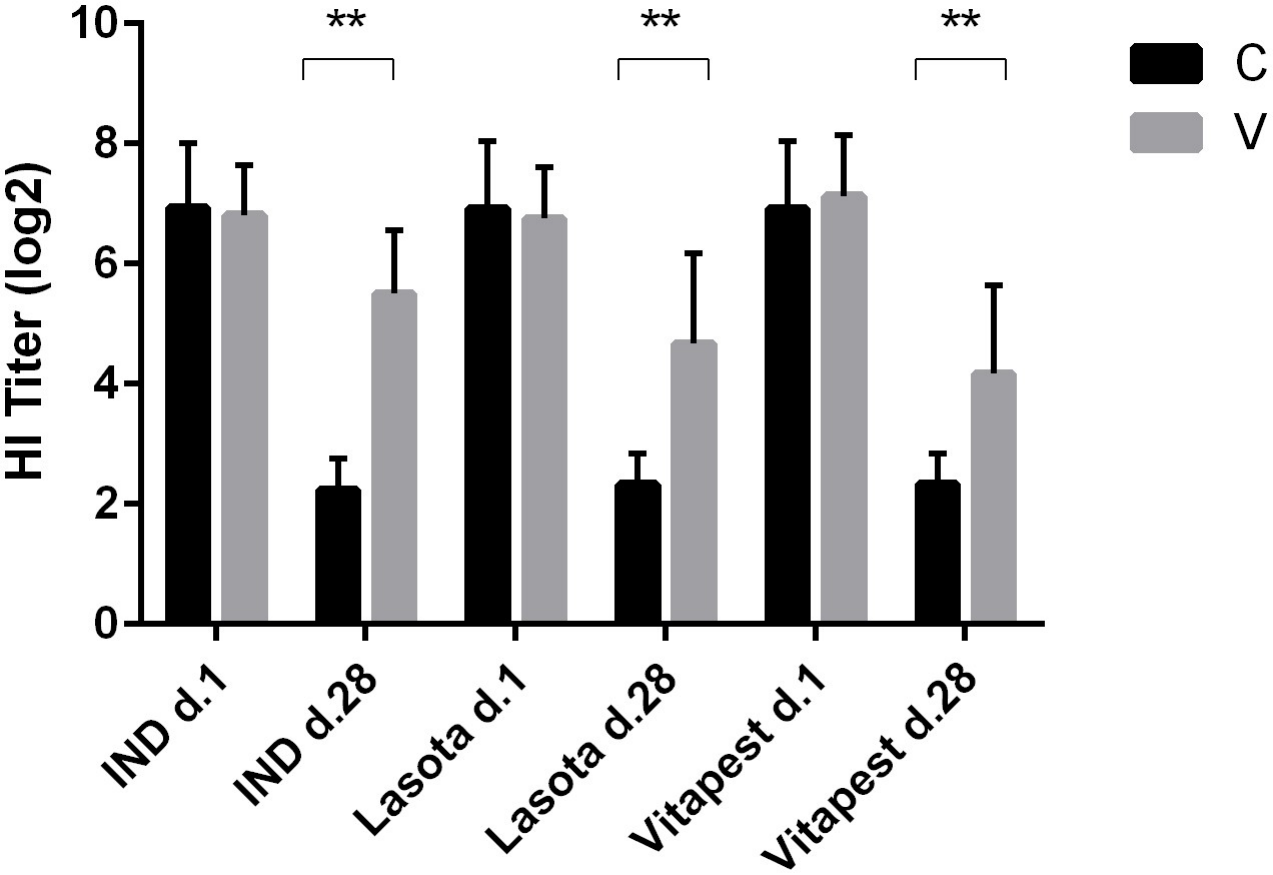
Antibodies levels in day old chicks (d.1) and 28 d in the control (C) and vaccinated (V) groups (n = 10 per group). Antibody was measured by Haemagglutination-Inhibition test and mean titer expressed as log 2 of serum serial dilution. Statistical differences between groups indicated as bars with star (** = *P ≤* 0.01).

Fig 2 shows antibody titers against the vaccine in three vaccinated (n = 10 per group) and 2 control groups (n = 10 per group). HI titer in IND group was significantly higher than Lasota and Vitapest. Although there was a significant difference between both Lasota and Vitapest and the controls, no statistically significant difference was observed between Lasota versus Vitapest group.

**Fig 3:**
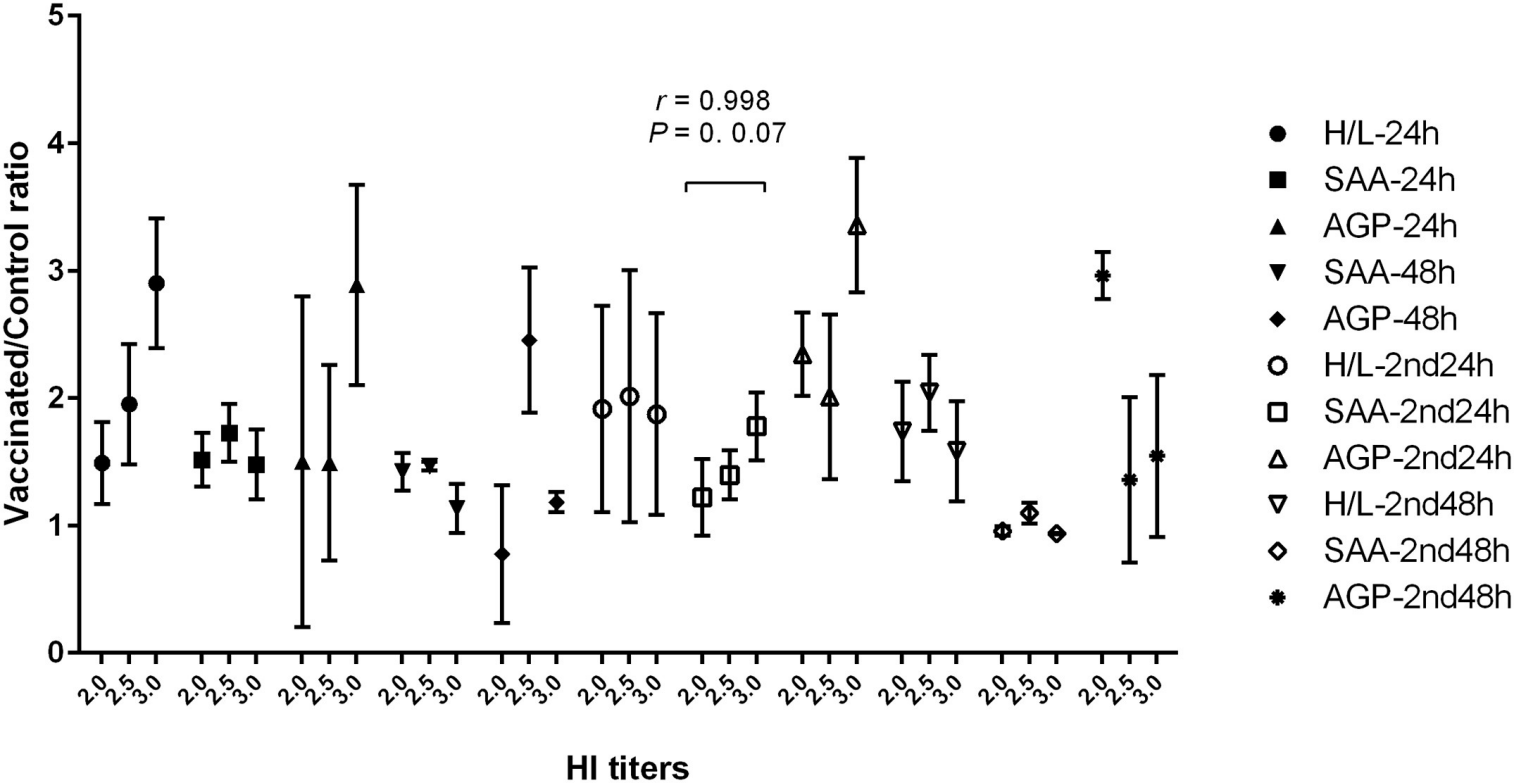
Correlations between HI titers and the H/L or APPs values. All data were expressed as ratio (ratio of the vaccinated to control groups). The results show a strong positive correlation between HI titers and SAA values after second vaccination (SAA-2nd24h).

Analyzing the correlations between HI titers and APR values at different specific time points, indicated a significant positive correlation between SAA values on day 22 (24h post second vaccination) and HI titers on day 28 (*r* = 0.998, *P* ≤ 0.05). No other significant correlations to HI titer were found (Fig 3).

## Discussion

Broiler chickens experience an acute-phase response through vaccination, which reflects the innate immunity and stress related to immunization. It is also considered that APR can modulate adaptive immunity and response to infection. Assessment of APR reaction by means of APPs can assist monitoring the vaccination stress and optimizing animal health. Our hypothesis stated that different type of vaccinations may result in alterations in the acute-phase responses which in turn might be related to humoral immunity and antibody response. For this purpose the heterophil-lymphocyte ratios and the concentrations of major acute phase proteins (SAA and AGP) in broiler chickens in response to three ND vaccines were investigated. The H/L ratio before vaccination (0.57 ± 0.13) was higher than previously reported for layer chicks (0.2 ± 0.12) [8]. In comparison between three vaccines, H/L ratio dramatically increased to 3 fold (1.48 ± 0.41) in IND group while the Lasota and Vitapest groups showed a milder response (Table 1). This is not surprising considering that IND can cause a local reaction, but the difference between Lasota and Vitapest vaccinated birds by the same ocular route, most likely is related to virus strain and its ability for replication and induction of inflammation.

SAA, like most species, is a major APP in chicken that plays an important role in host defense and immune modulation [11]. Our results indicated that SAA concentration of 9 days old broiler chicks (0.066 ± 0.012 mg/L) is close to values reported for the layer chicks (0.063 mg/mL), immunized by a combined ND and infectious bronchitis vaccine [8]. After ND vaccination, SAA level immediately increased to 2 fold (0.100 to 0.114 mg/L), but still less than that of the layer chicks (0.181 mg/mL) [8]. According to the literature data, basal levels of chicken SAA varied from 0.16 in layers to 1.5 mg/L in broilers [11–13]. In vaccinated birds, after 24h, the concentration of SAA increased by 2-fold in Lasota group (0.14 ± 0.002) and 1.8-fold in IND group (0.116 ± 0.015). Interestingly, a significant change (0.113 ± 0.016) in Vitapest group was detected 48 hours after the first vaccination and remained different from control OC until the second vaccination (Fig 1). A sharp and significant elevation of SAA in IND group, compared to Lasota and Vitapest, may reflect the role of SAA in immune modulation against live strains and their replication behaviors. It could be speculated that responses to killed vaccine, like IND group, are mainly caused by local inflammation due to the adjuvant and intramuscular injection.

Alpha 1-acid glycoprotein, as a moderate APP and a natural anti-inflammatory agent, is commonly used to evaluate acute phase response and immunity of chickens. In this experiment AGP drastically increased from 312.19 ± 147.4 mg/L to 3.5-fold (1082.3 ± 295.1) one day after IND vaccination. Basal level of AGP in broiler is approximately similar to the layer chicks (470 ± 170 mg/L) [8;11]. Elevation pattern of AGP in IND and Vitapest groups after the first vaccination was different from Lasota. In birds vaccinated by Lasota, AGP increased after 24h and reached a peak of 1034.9 ± 589.5 after 48h. A similar pattern for SAA was also found after second immunization in all three groups. In comparison with the other groups, it seems that Lasota had an immediate and long lasting response of SAA and AGP after first vaccination (Fig 1).

The humoral immune response to three vaccines was compared 7 days after the second vaccinations (day 28) (Fig 2). IND vaccination introduced a stronger immune response (5.50 ± 1.05) while the Vitapest had the lowest response (4.17 ± 1.47). Although all vaccinated birds had highly significant elevation in their HI titers, compared to the controls, the differences in IND vs. Lasota and Lasota vs. Vitapest were not statistically significant. As demonstrated by H/L ratio and APPs results, type of vaccine can alter the APP reaction and the level of humoral response in ND vaccination. However, when comparing both Lasota and Vitapest, there are minor differences in antibody production. It has been also anticipated that birds with high H/L ratios produce lesser antibody due to stress-induced suppression [14]. This is likely correct in cases of chronic inflammatory states. Although the IND group with the highest H/L ratio shows an increase in antibody production, the correlation analysis merely indicates a significant positive correlation between SAA concentrations and HI titers. Changes in H/L ratios, as a parameter of stress in poultry, reflect the innate immune response but not exactly the consequence of specific humoral response. Agonistic (stimulation) or antagonistic (suppression) cross-regulation between stress/APR and adaptive immunity might be related to the type of vaccine and the state of inflammation [14]. In our experiment, a positive correlation between SAA concentration and HI titer is indicative of agonistic cross regulation.

In conclusion, we monitored APR in response to three commercial ND vaccines that are routinely used for immunization of broiler chickens. A limitation of this experiment was that the administration route could not be compared due to the lack of adjuvant control in the IND group. Oil emulsions, preservatives and the type of adjuvant preparations can result in alterations in induction of APRs. However, it could be demonstrated that the post vaccine APPs response varies according to the type of vaccine. Our results also suggest the importance of SAA in broiler chicken, its correlation to the specific antibody titer and its potential use as a biomarker for adaptive immunity. Data generated from this work can be used for monitoring the current vaccination schedules as well as the next-generation vaccine design that needs the special safety considerations. However, while the inflammation/APRs are important protective mechanisms, in some levels and durations they can lead to a pathological state. Further clinical investigations are needed to determine the critical levels of APPs that are related to damage or protection.
