# Correction: Effects of vaccination on acute-phase protein response in broiler chicken

**DOI:** 10.1371/journal.pone.0230949

**Published:** 2020-03-19

**Authors:** Arash Janmohammadi, Nariman Sheikhi, Hadi Haghbin Nazarpak, Gholamreza Nikbakht Brujeni

There is an error in affiliation 2 for the third author Hadi Haghbin Nazarpak. The correct affiliation 2 is: Department of Clinical Science, Faculty of Veterinary Medicine, Garmsar Branch, Islamic Azad University, Garmsar, Iran.
